# Exercise and Nutrition Prehabilitation Program During Preoperative Chemotherapy Followed by Esophagectomy in Older Patients With Esophageal Cancer: A Randomized Clinical Trial

**DOI:** 10.1002/ags3.70127

**Published:** 2025-12-08

**Authors:** Keijiro Sugimura, Takashi Kanemura, Tomohira Takeoka, Takahito Sugase, Norihiro Matsuura, Kazuyoshi Yamamoto, Masahiko Yano, Hiroshi Miyata

**Affiliations:** ^1^ Department of Gastroenterological Surgery Osaka International Cancer Institute Osaka Japan; ^2^ Department of Surgery Kansai Rosai Hospital Amagasaki Japan; ^3^ Department of Surgery Sakai City Medical Center Sakai Japan

**Keywords:** esophageal cancer, older, prehabilitation, preoperative chemotherapy

## Abstract

**Background:**

We conducted a single‐center randomized prospective phase 2 trial to investigate whether exercise alone or prehabilitation intervention, including exercise and nutrition, is effective in increasing skeletal muscle mass during neoadjuvant chemotherapy for elderly esophageal cancer patients.

**Methods:**

Patients aged ≥ 60 years scheduled for preoperative chemotherapy followed by esophagectomy were randomized into three groups: Group A (no intervention), Group B (exercise‐only), and Group C (exercise plus nutritional intervention). Interventions occurred before treatment and surgery. The primary outcome was changes in skeletal muscle mass during chemotherapy.

**Results:**

Among the 99 patients enrolled, 88 were analyzed: 31 in Group A, 26 in Group B, and 31 in Group C. The skeletal muscle mass decreased by 1.1% in Group A, increased by 0.9% in Group B, and increased by 1.7% in Group C. The change in skeletal muscle mass in Group C was significantly higher than that in Group A (*p* = 0.013). The change in skeletal muscle mass in Group B tended to be higher than Group A, but the difference did not reach significance (*p* = 0.140) Group C showed a greater increase in body weight, skeletal muscle mass index, and gait speed than Group A (*p* = 0.014, *p* = 0.044, and *p* = 0.031, respectively). In Group B, skeletal muscle mass and skeletal muscle index tended to increase, but did not reach statistical significance compared to Group A (*p* = 0.140, *p* = 0.096).

**Conclusions:**

Prehabilitation, including nutrition and exercise, is effective in increasing skeletal muscle mass during neoadjuvant chemotherapy for older patients with esophageal cancer.

**Trial Registration:**

Japan Registry of Clinical Trials: jRCT s051190016

## Graphical abstract

This prospective randomized trial evaluated whether prehabilitation, exercise and nutrition intervention, enhance skeletal muscle mass during neoadjuvant chemotherapy in older patients with esophageal cancer undergoing esophagectomy. We also investigated whether exercise alone was sufficient to increase skeletal muscle mass, and whether nutritional support was necessary. The results showed that exercise alone was insufficient to increase skeletal muscle mass and that combining exercise with nutrition was essential and that prehabilitation improved walking speed during preoperative chemotherapy. This study is the first to demonstrate that exercise alone is insufficient and that exercise plus nutritional prehabilitation is essential during NAC in older patients with esophageal cancer.

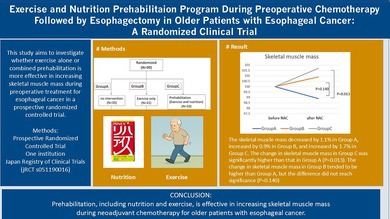

## 1 Introduction

Esophageal cancer is a common gastrointestinal malignancy and is among the most lethal carcinomas [1]. Given the limited efficacy of surgery alone, multimodal treatments—such as preoperative chemotherapy and chemoradiotherapy—have been adopted [2]. The CROSS trial established neoadjuvant chemoradiotherapy as the standard treatment for resectable advanced esophageal cancer in Western countries [3, 4]. The ESOPEC trial showed that neoadjuvant chemotherapy (NAC) using the FLOT regimen is a promising approach that is expected to improve outcomes [5]. NAC with CF plus docetaxel (DCF) regimens has shown high response rates and favorable long‐term prognoses [6, 7].

Sarcopenia is defined as the progressive loss of skeletal muscle mass and function due to aging or other diseases [8]. Sarcopenia is associated with chemotherapy‐related toxicities and may reduce treatment efficacy [9]. It is also linked to perioperative complications and poor survival in various solid cancers [10]. In esophageal cancer, sarcopenia is correlated with poor prognosis, especially in older patients [11, 12, 13, 14]. Furthermore, skeletal muscle mass often decreases during preoperative chemotherapy for esophageal cancer [13, 15]. Managing sarcopenia during preoperative treatment is essential for improving outcomes, but optimal supportive strategies to preserve skeletal muscles remain undefined.

Prehabilitation, combining preoperative nutrition and exercise, has effectively increased skeletal muscle mass in cancers such as gastric, colorectal, hepatocellular, bile duct, and pancreatic cancer [16, 17, 18, 19]. In esophageal cancer, prehabilitation may increase physical activity and potentially reduce the incidence of postoperative pneumonia [20, 21]. However, few prospective studies have investigated the effectiveness of prehabilitation during preoperative treatment for esophageal cancer. Two randomized control trials (RCTs) reported that prehabilitation during preoperative treatment improved NAC tolerance to NAC and grip strength [22, 23]. Whether prehabilitation increases skeletal muscle mass during preoperative treatment for esophageal cancer remains unclear. It is also unclear whether exercise alone is sufficient or if combined prehabilitation with nutrition is needed to increase skeletal muscle mass.

This study aims to investigate whether exercise alone or combined prehabilitation is more effective in increasing skeletal muscle mass during preoperative treatment for esophageal cancer in a prospective randomized controlled trial.

## 2 Patients and Methods

### 2.1 Study Design

This prospective randomized controlled trial was conducted at the Osaka International Cancer Institute, approved by its Ethics Committee for Clinical Research, and registered with the Japan Registry of Clinical Trials (jRCT s051190016). This study was conducted in accordance with the principles of the Declaration of Helsinki. Written informed consent was obtained from all participants.

### 2.2 Patients' Selection

Eligible patients were aged 60–85 years with histologically confirmed cT1b–T4 thoracic esophageal cancer, including M1 cases with supraclavicular lymph node metastases [24]. Additional criteria included Eastern Cooperative Oncology Group performance status 0–2, adequate organ function, and planned preoperative chemotherapy followed by radical surgery. Patients who underwent preoperative chemoradiotherapy were excluded. Only those who were able to consume food orally were included, although nasogastric supplementation was permitted for tumor‐related stenosis.

### 2.3 Randomization

Patients were randomized 1:1:1 to Group A (standard care), Group B (standard care + exercise program), or Group C (standard care + exercise program +nutritional therapy) using computer‐generated randomization (sequential permuted blocks of four) stratified by sex and clinical stage. Allocation concealment was maintained.

### 2.4 Intervention

#### 2.4.1 Standard Care

Baseline assessments were performed within 1 week of randomization, followed by immediate initiation of the intervention. All patients were reassessed 1 week before the surgical procedure. Standard care included physical activity recommendations, nutritional counseling, smoking cessation, and alcohol intake reduction. Malnourished patients were counseled by a registered dietitian. All patients wore pedometers to track their daily step count.

#### 2.4.2 Exercise Program

The exercise program included walking (> 7500 steps/day or ~1 h), handgrip training (10–20 kg, 20 repetitions/hand/day), and resistance training (standing, knee extension, hip flexion, hip abduction, and heel lifts). Specific exercises are shown in figures (Figure S1). Each resistance exercise was performed in three sets of 10 repetitions daily. Exercises were initiated under the supervision of a physical therapist during hospitalization for chemotherapy, with follow‐up checks conducted as needed during preoperative treatment.

#### 2.4.3 Nutritional Program

Patients in the nutrition program group received 250 mL/day (320 kcal/day) of branched‐chain amino acid‐rich nutritional supplements (Rehadays, Otsuka Pharmaceutical Factory Inc., Tokushima, Japan) (Figure S2). Each 125 mL pack contained 11.0 g protein, 3.4 g branched‐chain amino acids (including 2.3 g leucine), 2.2 g fat, and 24 g carbohydrates. Supplements were initiated immediately after the baseline assessment and continued until surgery.

### 2.5 Neoadjuvant Chemotherapy and Surgery

NAC included cisplatin‐fluorouracil (CF) or DCF. CF therapy comprised an 80 mg/m^2^ dose of cisplatin via drip infusion on day 1 and an 800 mg/m^2^ dose of 5‐FU via continuous infusion on days 1–5 [6]. DCF therapy comprised a 70 mg/m^2^ dose of docetaxel and cisplatin by drip infusion on day 1 and a 700 mg/m^2^ dose of 5‐FU by continuous infusion on days 1–5 [7]. Two chemotherapy courses were administered at a 3‐week interval. NAC was discontinued if the tumor did not shrink or if severe adverse events occurred during the first treatment course. Because this clinical trial was conducted before the results of JCOG1109 were published, the dose of the DCF regimen was based on the dose used in previous clinical trials conducted by our group [7]. Patients eligible for curative resection underwent surgery 3–6 weeks after chemotherapy. The patients underwent subtotal esophagectomy with two‐ or three‐field lymphadenectomy with curative intent via a right thoracotomy or thoracoscopic approach [25, 26]. Regional lymphadenectomy involved mediastinal, perigastric, and celiac nodes; distant lymphadenectomy included cervical lymph nodes.

### 2.6 Evaluation

Skeletal muscle mass (SMM) was assessed using multifrequency bioelectrical impedance analysis with eight electrodes (InBody 720; Biospace) [27]. Parameters measured included body weight, body mass index, total and appendicular SMM, and body fat mass. The appendicular SMM was calculated as the sum of the SMMs of the four limbs. Skeletal muscle mass index (SMI) was calculated as appendicular SMM/height [2]. Gait speed and 6‐min walk tests were performed according to the American Thoracic Society Committee on Proficiency Standards for Clinical Pulmonary Function Laboratories. Briefly, patients were instructed to walk a predetermined course at their own pace for 6 min. Standardized encouragement was provided to the patients every minute during the test. At 6 min, patients stopped walking, and the distance they walked was recorded. The 6‐min walk distance (MWD) was measured by a physical therapist in the Department of Rehabilitation.

### 2.7 Adverse Events and Effects of Preoperative Chemotherapy and Postoperative Complications

Acute adverse events were graded using the Common Terminology Criteria for Adverse Events, version 4.0. Surgical complications were assessed using the Clavien–Dindo classification [28]. We defined patients with Grade 2 complications as having complications. The pathological stage was determined according to the seventh edition of the Union Against Cancer classification system. The histological response was graded according to the criteria of the Japanese Society for Esophageal Diseases. Viable residual tumor cells within the entire tumor were graded as follows: Grade 3, no viable residual tumor cells; Grade 2, few residual tumor cells; Grade 1b, fewer than two‐thirds of residual tumor cells; Grade 1a, more than two‐thirds of residual tumor cells; and Grade 0, no significant response to preoperative treatment.

### 2.8 Outcomes

The primary endpoint was a change in skeletal muscle mass during preoperative chemotherapy. The secondary endpoints were the toxicity of preoperative chemotherapy, surgical complications, grip strength, gait speed, 6 MWD, body composition, and nutritional indicators, including body weight, albumin, and lymphocyte count.

### 2.9 Statistical Analysis

As the primary analysis, the change in skeletal muscle mass before and after preoperative chemotherapy was compared. We hypothesized a skeletal muscle mass loss of −7% in the control group and −3% in the intervention group. For a power of 90% with a 20% significance level, 0.05 alpha error, and 10% dropout, 30 patients per group were initially targeted. The final sample size is set at 90 patients in total. Group A, the control group, was compared with Group B, the intervention group, and if a significant level was reached, B was deemed to be the effective treatment. Group A, the control group, was compared with Group C, the intervention group, and if a significant level was reached, C was deemed to be the effective treatment. If both of the above analyses reached a significant level, groups B and C were compared, and if a significant level was reached, the treatment with the greater increase in skeletal muscle mass was deemed to be the effective treatment; but if a significant level was not reached, both B and C were deemed to be effective treatments. Statistical analyses were performed using the Statistical Package for Social Sciences version II software (SPSS Japan, Tokyo, Japan). Continuous variables are presented as mean ± standard deviation; categorical variables are expressed as numbers (percentages). Fisher's exact test and Student's *t*‐test were used to compare categorical and continuous variables, respectively.

## 3 Results

### 3.1 Patients Characteristics

**FIGURE 1:**
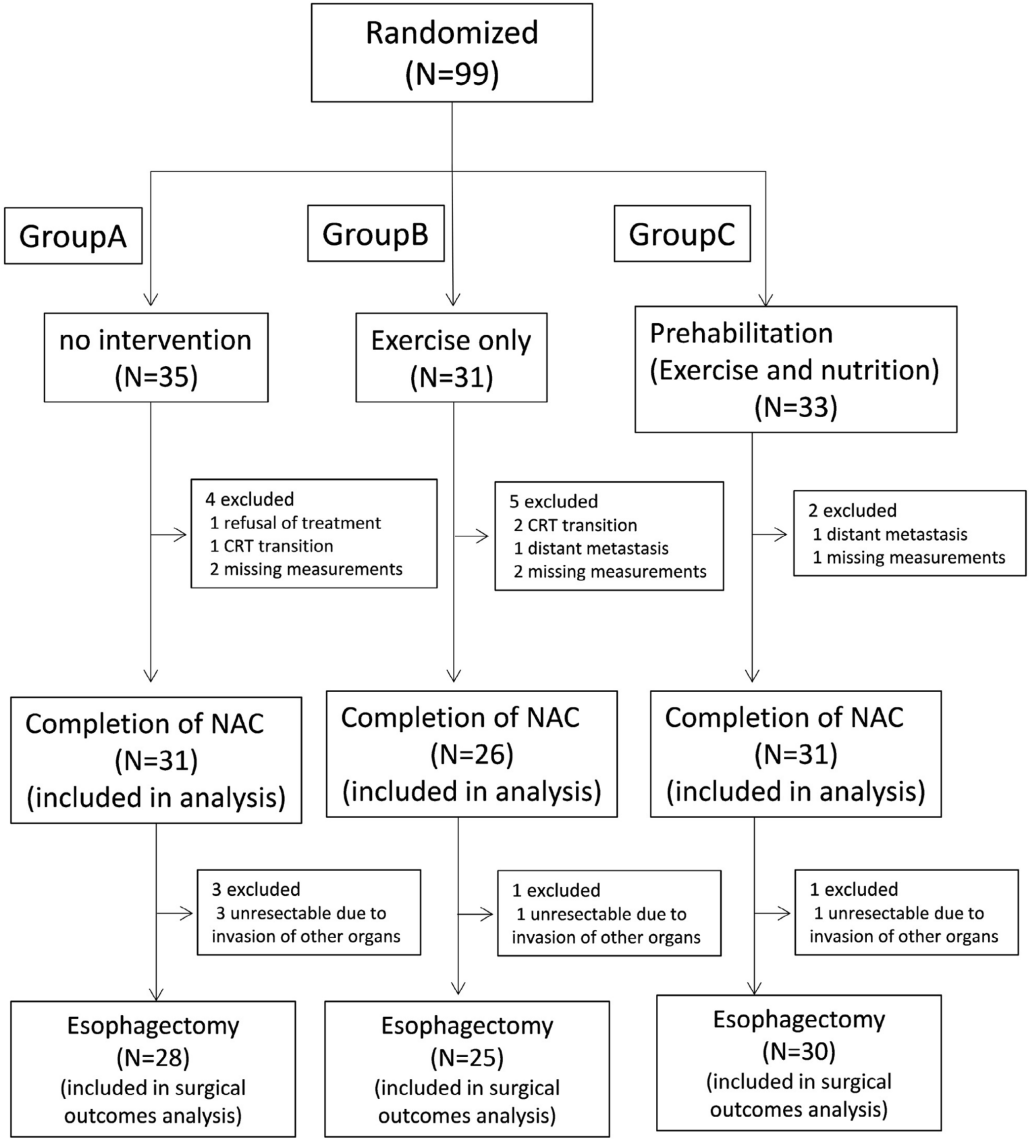
CONSORT diagram.

Ninety‐nine patients were included in this study between June 2019 and September 2023. Among the 99 eligible patients, 35 were randomly assigned to the control group (Group A), 31 to the exercise‐only group (Group B), and 33 to the exercise and nutritional support group (Group C). Among the patients assigned to each group, 11 were excluded from the analysis. The reasons for exclusion were refusal of treatment in one case, transition from preoperative chemotherapy to CRT in three cases, emergence of distant metastasis during treatment in two cases, and missing measurement data in five cases. Finally, 31 patients in Group A, 26 in Group B, and 31 in Group C were included in the analysis (Figure 1). The baseline characteristics were well balanced among the groups (Table 1). There were no statistically significant differences in age, sex, or oncological factors between the three groups. There were also no statistically significant differences in weight loss, dysphagia score, or oral intake at the time of initial examination between the three groups.

**TABLE 1:** Patient characteristics.

		Group A (*n* = 31)	Group B (*n* = 26)	Group C (*n* = 31)	*p* (A vs. B)/*p* (A vs. C)
Age		68.4 ± 4.4	70.7 ± 4.5	67.8 ± 5.5	0.057/0.651
Sex	Male	24 (77%)	22 (85%)	24 (77%)	0.738/1.000
	Female	7 (23%)	4 (15%)	7 (23%)	
Height	(cm)	164.1 ± 8.5	164.0 ± 7.7	164.5 ± 6.4	0.955/0.854
Body weight	(kg)	57.8 ± 11.2	55.0 ± 8.7	58.2 ± 12.1	0.297/0.887
BMI	(kg/m^2^)	21.3 ± 3.2	20.4 ± 2.8	21.4 ± 4.0	0.250/0.905
Performance status	0	30 (97%)	24 (92%)	30 (97%)	0.587/1.000
	1	1 (3%)	2 (8%)	1 (3%)	
Comorbidity	Hypertension	8 (26%)	7 (27%)	8 (26%)	1.000/1.000
	Cardiac disease	2 (6%)	6 (23%)	3 (10%)	0.124/1.000
	COPD	2 (6%)	3 (12%)	0 (0%)	0.651/0.492
	DM	3 (10%)	4 (15%)	3 (10%)	0.691/1.000
	Vascular disease	1 (3%)	1 (4%)	3 (10%)	1.000/0.612
Dysphagia score	0	5 (16%)	2 (8%)	7 (23%)	0.244/0.375
	1	18 (58%)	14 (54%)	19 (61%)	
	2	8 (26%)	8 (31%)	4 (13%)	
	3	0 (0%)	2 (8%)	1 (3%)	
	4	0 (0%)	0 (0%)	0 (0%)	
Body weight loss at first visit	(kg)	−2.1 ± 2.7	−2.4 ± 2.8	−1.6 ± 2.7	0.643/0.516
Histology	SCC	30 (97%)	25 (96%)	29 (94%)	1.000/1.000
	Adenocacrcinoma	1 (3%)	1 (4%)	2 (6%)	
Location	Upper	9 (29%)	6 (23%)	12 (39%)	0.822/0.472
	Middle	19 (61%)	18 (69%)	18 (58%)	
	Lower	3 (10%)	2 (8%)	1 (3%)	
Clinical T stage	cT1	1 (3%)	0 (0%)	2 (6%)	0.464/0.798
	cT2	5 (16%)	3 (12%)	4 (13%)	
	cT3	25 (81%)	23 (88%)	25 (81%)	
Clinical N stage	cN0	6 (19%)	10 (38%)	8 (26%)	0.256/0.519
	cN1	19 (62%)	13 (50%)	20 (65%)	
	cN2	6 (19%)	3 (12%)	3 (10%)	
Clinical M stage	cM0	29 (94%)	26 (100%)	31 (100%)	0.495/0.492
	cM1 (lym)	2 (6%)	0 (0%)	0 (0%)	
Clinical stage	cStage1	1 (3%)	0 (0%)	2 (6%)	0.285/0.501
	cStage2	10 (32%)	13 (50%)	10 (32%)	
	cStage3	18 (58%)	13 (50%)	19 (61%)	
	cStage4	2 (6%)	0 (0%)	0 (0%)	
Laboratory data	Hemoglobin (g/dL)	13.4 ± 1.6	13.7 ± 1.3	13.8 ± 1.6	0.836/0.306
	Albumin (g/dL)	4.0 ± 0.4	3.8 ± 0.5	3.9 ± 0.4	0.111/0.444
	Lymphocyte (/μL)	1401 ± 486	1350 ± 376	1350 ± 376	0.757/0.644
	CRP (mg/dL)	0.4 ± 0.6	0.8 ± 1.2	0.4 ± 0.7	0.211/0.747
Oral intake before treatment	(kcal)	1133 ± 379	1125 ± 372	1232 ± 345	0.952/0.623
Skeletal muscle mas	(kg)	24.1 ± 4.9	23.4 ± 3.9	25.1 ± 5.0	0.718/0.236
SMI	(kg/m^2^)	31.5 ± 7.9	30.7 ± 6.0	33.1 ± 6.1	0.686/0.400
Handgrip strength	(kg)	31.5 ± 7.9	30.7 ± 6.0	33.1 ± 6.1	0.926/0.368
Gait speed	(m/s)	1.36 ± 0.25	1.37 ± 0.19	1.42 ± 0.21	0.935/0.164
6MWD	(m)	457 ± 79	459 ± 66	485 ± 65	0.533/0.164

Abbreviations: 6MWD, 6‐min walk distance; BMI, body mass index; COPD, chronic obstructive pulmonary disease; CRP, C‐reactive protein; DM, diabetes mellitus; lym, lymphocytes; SCC, squamous cell carcinoma.

### 3.2 Exercise and Nutritional Intervention

The mean duration of the exercise and nutritional intervention program was 8.7 weeks. The preoperative chemotherapy regimen used was DCF in all patients (Table 2). Eighty‐four patients (95%) received two cycles of preoperative chemotherapy. One patient in Group A and three patients in Group C completed preoperative chemotherapy after one cycle owing to insufficient efficacy or adverse events. The intervention began a median of 1 day (range 0–4 days) before the start of preoperative chemotherapy. Measurements after intervention, including body composition and physical activity, were performed a median of 30 days (range 14–85 days) after the completion of preoperative chemotherapy. Esophagectomy was performed a median of 4 days after the measurements. The median duration of intervention during preoperative chemotherapy was 59 days (range 41–145 days), of which the median length of hospital stay was 28 days (19–52 days). Adherence to the intervention during hospitalization and after discharge was assessed by having each patient keep a record of their rehabilitation and nutritional medication and by doctors' checking the record regularly. The mean daily step counts were 3022, 3864, and 4638 steps per group, respectively. The number of steps taken by Group B was comparable to that of Group A, whereas Group C took significantly more steps than Group A (A vs. B; *p* = 0.161, A vs. C, *p* = 0.019). The mean number of hand grip repetitions was 25.6 in Group B and 32.4 in Group C. The average number of each exercise performed in Group B was 20 stand‐ups, 20 knee bends, 20 hip joint openings, and 20 heel lifts. The average number of each exercise performed in Group C was 23 stand‐ups, 23 knee bends, 23 hip joint openings, and 24 heel lifts. The average amount of nutritional supplements consumed in Group C was 0.89 bottles (111 mL). The median duration of intervention during preoperative chemotherapy was 59 days (range 41–145 days), of which the median length of hospital stay was 28 days (19–52 days).

**TABLE 2:** Neoadjuvant chemotherapy and adherence to the prehabilitation program.

	Group A (*n* = 31)	Group B (*n* = 26)	Group C (*n* = 31)	*p* (A vs. B)/*p* (A vs. C)
Regimen of chemotherapy				
DCF	31 (100%)	26 (100%)	31 (100%)	1.000/1.000
CF	0	0	0	
Cycle				
1	1 (3%)	0 (0%)	3 (10%)	1.000/0.612
2	30 (97%)	26 (100%)	28 (90%)	
Daily step count (step) (adherence, %)	3022 ± 1469	3864 ± 210 (55.2%)	4638 ± 2519 (66.2%)	0.161/0.019
Hand grip (times) (adherence, %)	—	25.6 ± 17.4 (128%)	32.4 ± 18.4 (162%)	—
Exercise				
Stand up (times)	—	20 ± 9	23 ± 17	—
Knee bending (times)		20 ± 9	23 ± 17	—
Knee lift (times)		20 ± 9	23 ± 17	—
Hip joint opening (times)		20 ± 9	23 ± 17	—
Heel lift (times) (adherence, %)		20 ± 9 (66.7%)	24 ± 17 (77.3%)	—
Nutrition (bottle/day) (adherence, %)	—	—	0.89 ± 0.49 (44.5%)	—

Abbreviations: CF, cisplatin‐fluorouracil; DCF, cisplatin‐fluorouracil plus docetaxel.

### 3.3 Adverse Events and Effects of Preoperative Chemotherapy

There were no differences in hematological toxicity among the three groups (Table 3). In non‐hematological toxicity, the incidence of febrile neutropenia was 68% in Group A, 46% in Group B, and 39% in Group C. The incidence of febrile neutropenia was lower in Group C than in Group A; however, the difference was not statistically significant. There were no other differences in non‐hematological toxicity, including diarrhea, oral mucositis, nausea, appetite loss, and creatinine increase, among the three groups.

**TABLE 3:** Chemotherapy‐related adverse events.

		Group A (*n* = 31)	Group B (*n* = 26)	Group C (*n* = 31)	*p* (A vs. B)/*p* (A vs. C)
Hematological toxicity					
Leukopenia	Grade0–2	3 (10%)	4 (15%)	6 (19%)	0.691/0.473
	Grade3–4	28 (90%)	22 (85%)	25 (81%)	
Neutropenia	Grade0–2	2 (6%)	1 (4%)	2 (6%)	1.000/1.000
	Grade3–4	29 (94%)	25 (96%)	29 (94%)	
Lymphopenia	Grade0–2	18 (58%)	16 (62%)	24 (77%)	1.000/0.174
	Grade3–4	13 (42%)	10 (38%)	7 (23%)	
Anemia	Grade0–2	30 (97%)	26 (100%)	29 (94%)	1.000/1.000
	Grade3–4	1 (3%)	0 (0%)	2 (6%)	
Thrombocytopenia	Grade0–2	31 (100%)	26 (100%)	31 (100%)	1.000/1.000
	Grade3–4	0 (0%)	0 (0%)	0 (0%)	
Nonhematological toxicity					
Febrile neutropenia	Grade0	10 (32%)	14 (54%)	19 (61%)	0.115/0.073
	Grade3–4	21 (68%)	12 (46%)	12 (39%)	
Diarrhea	Grade0–1	13 (42%)	15 (58%)	19 (61%)	0.292/0.204
	Grade2–4	18 (58%)	11 (42%)	12 (39%)	
Oral mucositis	Grade0–1	30 (97%)	25 (96%)	28 (90%)	1.000/0.612
	Grade2–4	1 (3%)	1 (4%)	3 (10%)	
Nausea	Grade0–1	31 (100%)	25 (96%)	31 (100%)	0.456/1.000
	Grade2–4	0 (0%)	1 (4%)	0 (0%)	
Appetite loss	Grade0–1	28 (90%)	25 (96%)	27 (87%)	0.617/1.000
	Grade2–4	3 (10%)	1 (4%)	4 (13%)	
Creatinine increased	Grade0–1	29 (94%)	25 (96%)	31 (100%)	1.000/0.492
	Grade2–4	2 (6%)	1 (4%)	0 (0%)	

### 3.4 Impact of the Exercise and Nutritional Intervention

**FIGURE 2:**
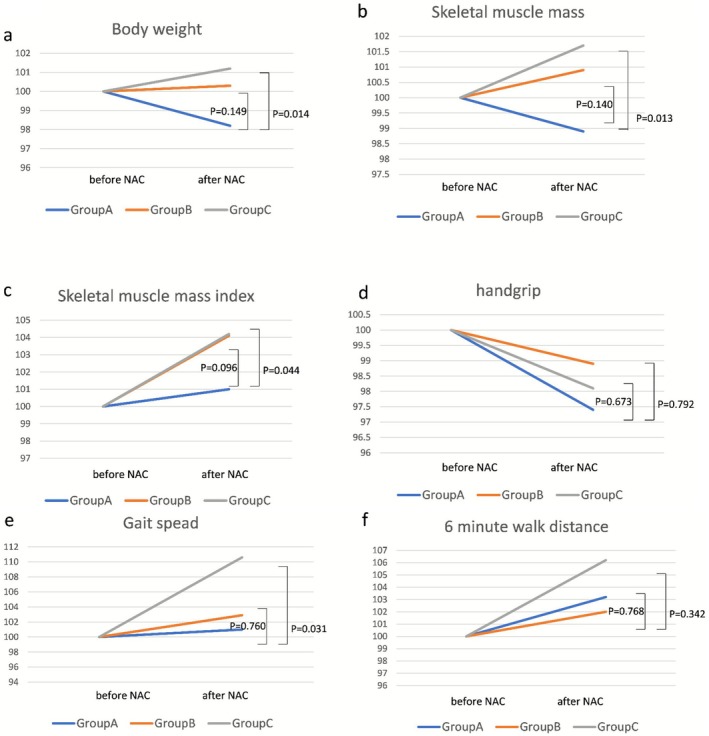
(a) Body weight change ratio during preoperative chemotherapy. The body weight change in Group B tended to be greater than that in Group A, and the difference was not statistically significant (*p* = 0.149). The body weight change in Group C was significantly greater than that in Group A (*p* = 0.014). (b) Skeletal muscle mass change ratio during preoperative chemotherapy. The skeletal muscle mass in Group B tended to be higher than that in Group A; however, the difference was not statistically significant (*p* = 0.140). The skeletal muscle mass in Group C was significantly higher than that in Group A (*p* = 0.013). (c) Skeletal muscle index change ratio during preoperative chemotherapy. The skeletal muscle index in Group B tended to be higher than that in Group A; however, the difference was not statistically significant (*p* = 0.096). The skeletal muscle index was significantly higher in Group C than in Group A (*p* = 0.044). (d) Handgrip strength change ratio during preoperative chemotherapy. There was no statistically significant difference in the rate of change in the handgrip strength change ratio during preoperative chemotherapy among the three groups. (e) Gait speed change ratio during preoperative chemotherapy. There was no statistically significant difference in the change rates of gait speed between Groups A and Group B. The gait speed change ratio in Group C was significantly higher than that in Group A (*p* = 0.031). (f) 6‐min walk distance change ratio during preoperative chemotherapy. There was no statistically significant difference in the rate of change in the minute walk distance during preoperative chemotherapy among the three groups (*p* = 0.768, *p* = 0.342).

Changes in body weight, body composition, grip strength, walking speed, and nutritional indicators during preoperative chemotherapy are shown in Figure 2 and Table S1. The rates of body weight change in groups A, B, and C were 98.2%, 100.3%, and 101.2%, respectively. The rate of increase in Group B was equivalent to that of Group A, but the rate of increase in Group C was significantly higher than that of Group A (A vs. B; *p* = 0.149, A vs. C; *p* = 0.014), and the rates of change in SMM in groups A, B, and C were 98.9%, 100.9%, and 101.7%, respectively. The rate of change in Group B was equivalent to that in Group A; however, the rate of increase in Group C was significantly higher than that in Group A (A vs. B; *p* = 0.140, A vs. C; *p* = 0.013). The rate of change in the SMI also tended to be similar in each group (A vs. B; *p* = 0.096, A vs. C; *p* = 0.044). The rate of change in grip strength during chemotherapy was 97.2%, 98.9%, and 98.1%, respectively, with similar results across the groups (A vs. B; *p* = 0.673, A vs. C; *p* = 0.792). The rate of change in gait speed was 101.0%, 102.9%, and 110.6%, respectively, in the respective groups. Groups A and B were comparable, but the rate of change in Group C was significantly greater than that in Group A (A vs. B; *p* = 0.760, A vs. C; *p* = 0.031), and the rates of 6 MWD were 103.2%, 102.0%, and 106.2%, respectively. The rate of change in the 6 MWD tended to be higher in Group C, but the difference was not statistically significant between groups (A vs. B; *p* = 0.768, A vs. C; *p* = 0.342). There was no difference in the rate of change in albumin or lymphocyte count during preoperative chemotherapy among the three groups.

### 3.5 Surgical Procedure, Pathological Findings, and Postoperative Complications

The surgical procedures and pathological findings are shown in Table 4. Subcutaneous reconstruction was performed in three patients (12%) in group B. The reasons for performing subcutaneous reconstruction in these three patients were as follows: two had undergone cardiac surgery, and one underwent two‐staged surgery because of poor respiratory function. The histological effect of the primary tumor was Grade 2 or higher in 36% of Group A, 36% of Group B, and 57% of Group C. The effect in Group C tended to be greater than that in Group A, and the difference was not statistically significant.

**TABLE 4:** Surgical procedures and histopathological findings.

		Group A (*n* = 28)	Group B (*n* = 25)	Group C (*n* = 30)	*p* (A vs. B)/*p* (A vs. C)
Thoracic approach	Thoracotomy	2 (7%)	2 (8%)	2 (7%)	1.000/1.000
	Thoracoscopy	26 (93%)	23 (92%)	28 (93%)	
Abdominal approach	Hand‐assisted	20 (71%)	19 (76%)	23 (77%)	0.763/0.767
	Laparoscopy	8 (29%)	6 (24%)	7 (23%)	
Node dissection	2‐field	11 (39%)	5 (20%)	6 (20%)	0.202/0.151
	3‐field	17 (61%)	19 (76%)	24 (80%)	
	Other	0 (0%)	1 (4%)	0 (0%)	
Reconstruction organ	Stomach	28 (100%)	25 (100%)	30 (100%)	1.000/1.000
Reconstruction route	Retrosternal	27 (96%)	21 (84%)	27 (90%)	0.166/0.380
	Posterior mediastinal	1 (4%)	1 (4%)	1 (3%)	
	Subcutaneous	0 (0%)	3 (12%)	2 (7%)	
Curability	R0	26 (93%)	23 (92%)	28 (93%)	1.000/1.000
	R1/2	2 (7%)	2 (8%)	2 (7%)	
ypT stage	ypT0	8 (29%)	0 (0%)	3 (10%)	0.057/0.091
	ypT1	4 (14%)	7 (28%)	9 (30%)	
	ypT2	3 (11%)	5 (20%)	9 (30%)	
	ypT3	11 (39%)	11 (44%)	7 (23%)	
	ypT4	2 (7%)	2 (8%)	2 (7%)	
ypN stage	ypN0	15 (54%)	10 (40%)	8 (27%)	0.798/0.299
	ypN1	6 (21%)	5 (20%)	10 (33%)	
	ypN2	4 (14%)	6 (24%)	7 (23%)	
	ypN3	1 (4%)	2 (8%)	3 (10%)	
	Unknown	2 (7%)	2 (8%)	2 (7%)	
ypM stage	ypM0	26 (93%)	25 (100%)	29 (97%)	0.395/0.802
	ypM1	2 (7%)	0 (0%)	1 (3%)	
ypStage	ypStage0	5 (18%)	0 (0%)	3 (10%)	0.114/0.303
	ypStage1	6 (21%)	4 (16%)	2 (7%)	
	ypStage2	5 (18%)	9 (36%)	11 (37%)	
	ypStage3	9 (32%)	10 (40%)	11 (37%)	
	ypStage4	1 (4%)	0 (0%)	1 (3%)	
	Unknown	2 (7%)	2 (8%)	2 (7%)	
Histological grade	Grade0	0 (0%)	0 (0%)	0 (0%)	0.021/0.003
	Grade1a	9 (32%)	7 (29%)	10 (33%)	
	Grade1b	7 (25%)	7 (29%)	1 (3%)	
	Grade2	2 (7%)	9 (36%)	14 (47%)	
	Grade3	8 (29%)	0 (0%)	3 (10%)	
	Unknown	2 (7%)	2 (8%)	2 (7%)	
	Grade0–1b	16 (62%)	14 (61%)	11 (39%)	1.000/0.173
	Grade2–3	10 (38%)	9 (39%)	17 (61%)	

Postoperative complication incidence was 43% in Group A, 40% in Group B, and 30% in Group C, with no significant differences among the three groups (Table 5). The incidence of postoperative pneumonia was 14% in Group A, 8% in Group B, and 3% in Group C. The incidence of postoperative pneumonia was lower in Group C than in Group A; however, the difference was not statistically significant (*p* = 0.187). The incidence of other postoperative complications was similar in all three groups.

**TABLE 5:** Postoperative complications.

	Group A (*n* = 28)	Group B (*n* = 25)	Group C (*n* = 30)	*p* (A vs. B)/*p* (A vs. C)
Any	12 (43%)	10 (40%)	11 (37%)	1.000/0.789
Pneumoniae	4 (14%)	2 (8%)	1 (3%)	0.672/0.187
Anastomotic leakage	1 (4%)	2 (8%)	1 (3%)	0.672/0.812
Recurrent laryngeal nerve palsy	2 (7%)	5 (20%)	3 (10%)	0.234/1.000
Cardiovascular	2 (7%)	2 (8%)	3 (10%)	1.000/1.000
Pneumothorax	1 (4%)	1 (4%)	0 (0%)	1.000/0.483
Chylothorax	0 (0%)	0 (0%)	0 (0%)	1.000/1.000
Venous thrombosis	0 (0%)	0 (0%)	0 (0%)	1.000/1.000
Surgical site infection	0 (0%)	1 (4%)	2 (7%)	0.472/0.492
Bleeding	1 (4%)	0 (0%)	0 (0%)	1.000/1.000
Others	2 (7%)	1 (4%)	4 (13%)	1.000/0.671
Reoperation	0 (0%)	2 (8%)	0 (0%)	0.218/1.000
In‐hospital mortality	0 (0%)	0 (0%)	0 (0%)	1.000/1.000
Postoperative Hospital stay (days, median)	15.5	18.0	15.5	0.074/0.286

## 4 Discussion

This prospective randomized trial evaluated whether prehabilitation enhances skeletal muscle mass during NAC in older patients with esophageal cancer undergoing esophagectomy. We also investigated whether exercise alone was sufficient to increase skeletal muscle mass, and whether nutritional support was necessary. The results showed that exercise alone was insufficient to increase skeletal muscle mass and that combining exercise with nutrition was essential. Prehabilitation also improved walking speed during preoperative chemotherapy. This study is the first to demonstrate that exercise alone is insufficient and that exercise plus nutritional prehabilitation is essential during NAC in older patients with esophageal cancer.

The results of this study showed that exercise alone was insufficient to increase skeletal muscle mass during NAC in older patients with esophageal cancer and that prehabilitation combined with nutrition was essential. Two previous RCTs have investigated changes in skeletal muscle mass during preoperative treatment for esophageal cancer using prehabilitation. Xu et al. reported a skeletal muscle mass of −2.0% in the control group versus −0.7% in the prehabilitation group during chemoradiotherapy, suggesting a trend toward muscle preservation, although not statistically significant [22]. Allen et al. conducted another RCT in patients with gastroesophageal cancer receiving prehabilitation during preoperative chemotherapy or chemoradiotherapy [23]. They examined skeletal muscle mass changes using computed tomography before and after preoperative chemoradiotherapy. According to their results, skeletal muscle mass declined by −11% in the prehabilitation group compared to −15.6% in the control group, indicating a significant reduction in loss. These studies also demonstrated that prehabilitation during preoperative treatment for esophageal cancer was effective in increasing the skeletal muscle mass. Although both studies compared control and prehabilitation groups, they did not clarify whether exercise alone was sufficient to increase skeletal muscle mass or whether prehabilitation, including nutritional intervention, was necessary. The results of our study demonstrated that exercise alone is insufficient during NAC in older patients with esophageal cancer and that a combination of exercise and nutrition is essential.

Our findings align with prior reports showing that exercise alone is insufficient and that combined nutritional intervention is necessary to increase skeletal mass in older adults. Fiatarone et al. were the first to prospectively examine whether nutrition and exercise alone are sufficient to increase muscle strength in physically frail older individuals or whether a combination is necessary [29]. Their results were the first to demonstrate that combining nutrition with exercise was more effective in increasing muscle strength than either intervention alone. Kim et al. conducted a prospective study in older women with sarcopenia to determine whether nutrition or exercise alone could increase skeletal muscle mass or whether a combination was necessary [30]. The results showed that neither nutrition nor exercise alone was sufficient to increase skeletal muscle mass, and that their combination was the most effective approach. Several previous reports have demonstrated the mechanism by which combining nutrition and exercise effectively increases skeletal muscle mass in older individuals [31, 32]. These reports indicate that the decline in skeletal muscle mass in older individuals is related to reduced muscle protein synthesis, and that the anabolic response to leucine‐rich amino acids is highly dependent on amino acid‐induced muscle protein metabolism in this population. In our study, combining a leucine‐rich nutritional supplement with exercise was the most effective way to increase skeletal muscle mass.

The results of this study showed that prehabilitation significantly increased gait speed during NAC. In this study, prehabilitation also increased the 6‐min walking distance, but the difference did not reach statistical significance. Previous reports have shown contradictory results regarding whether prehabilitation increases physical levels before and after NAC. Xu et al. reported that prehabilitation significantly increased the 6 MWD before and after NAC [22]. Allen et al. also examined physical activity using peak VO2 before and after NAC. Their results indicated that prehabilitation did not improve peak VO_2_ [23]. In contrast, Minella et al.'s prehabilitation intervention study of patients with esophagogastric cancer not receiving NAC, found that prehabilitation significantly increased 6 MWD before surgery [20]. In hospitalized NAC regimens, as in this study, the effect of prehabilitation on physical function may be limited because of reduced walking distance and restricted daily activities during prolonged stays.

Prehabilitation during NAC tended to reduce febrile neutropenia, possibly because of the protective effect of enteral nutrition against adverse events. Miyata et al. reported that, during preoperative chemotherapy for esophageal cancer [33], enteral nutrition reduced hematologic toxicity compared with intravenous nutrition. Our findings are consistent with these results. Most patients received the DCF triplet regimen, which offers a high response rate but is associated with frequent adverse events. Prehabilitation may help prevent adverse events during preoperative chemotherapy for esophageal cancer.

This study showed that prehabilitation during NAC for esophageal cancer tended to reduce postoperative pneumonia more effectively than exercise alone. A previous report by Ida et al. reported that a preoperative skeletal muscle loss in the esophagus was associated with postoperative pneumonia [13]. Our retrospective study also found that reduced physical activity, as measured by the 6 MWD, was significantly associated with postoperative pneumonia [27]. In this study, prehabilitation during NAC increased skeletal muscle mass and improved physical activity, including walking speed and 6 MWD, likely contributing to reduced postoperative pneumonia. In this study, skeletal muscle mass was the primary endpoint; the sample size may have been insufficient to detect a significant effect of prehabilitation on postoperative pneumonia.

The adherence rates in this study were 76% for the exercise program, 62% for walking steps, 160% for grip strength training, and 45% for nutritional supplementation, the latter being lower than expected. The adherence rates were comparable to those reported in previous RCTs of prehabilitation during preoperative treatment for gastroesophageal cancer. Previous RCTs have reported adherence rates of 68%–76% [22, 23], whereas a review by Ikeda et al. cited a range of 56%–76% [34, 35]. In this study, a single exercise program was implemented, but given the variability in physical activity among older patients, tailoring exercise intensity to individual capacity may enhance adherence to prehabilitation. Multilevel intensity programs and close monitoring could address barriers such as low baseline activity or poor motivation.

In the present study, the daily step count was significantly higher in Group C than in Group A. One possible explanation is that Group A patients were receiving conventional prescribed therapy, while Groups B and C were instructed to walk a target of 7500 steps per day. Another possible explanation is that Group C tended to experience fewer adverse events related to febrile neutropenia compared to the other groups, which may have resulted in a higher daily step count. Group C also performed hand grips and exercises more frequently each day than Group B. A possible explanation for the higher number of exercises in Group B compared to Group C may be due to awareness towards exercise. Participants were instructed to take the nutritional supplement in conjunction with exercises, and awareness of the pairing of nutritional supplement intake and exercises might have led to greater participation in the exercise program. Furthermore, the increased amount of exercise in Group C may have led to an increase in skeletal muscle mass and physical activity.

This study had some limitations. First, it was conducted at a single institution and had a small sample size. Second, adherence to the exercise program was satisfactory; however, compliance with nutritional supplementation was low. Increasing compliance with nutritional supplements requires medical staff to interview patients during the study and implement a continuous, multidisciplinary program to support adherence. Third, five patients were excluded from the analysis due to missing measurements, representing 5% of enrolled patients. Fourth, a long‐term evaluation was not performed. Preoperative muscle mass and physical performance may affect long‐term outcomes. Future analyses are needed to determine whether the intervention during NAC in this study improves long‐term outcomes.

In conclusion, prehabilitation—including nutrition and exercise intervention—is effective in increasing skeletal muscle mass during NAC for older patients with esophageal cancer. In contrast, exercise intervention alone is insufficient to increase skeletal muscle mass during NAC in older patients with esophageal cancer. Future large‐scale trials are necessary to determine whether prehabilitation during NAC improves the clinical and oncological outcomes.

## Author Contributions

**Keijiro Sugimura:** conceptualization, methodology, writing – original draft, writing – review and editing, investigation, visualization, project administration, formal analysis, data curation, software, funding acquisition. **Takashi Kanemura:** investigation, writing – original draft, writing – review and editing, methodology, data curation. **Tomohira Takeoka:** conceptualization, methodology, writing – original draft, writing – review and editing. **Takahito Sugase:** conceptualization, methodology, writing – original draft, writing – review and editing. **Norihiro Matsuura:** conceptualization, methodology, writing – original draft, writing – review and editing. **Kazuyoshi Yamamoto:** conceptualization, methodology, writing – original draft, writing – review and editing. **Masahiko Yano:** conceptualization, methodology, writing – original draft, writing – review and editing, supervision, funding acquisition. **Hiroshi Miyata:** conceptualization, methodology, supervision, writing – original draft, writing – review and editing, funding acquisition.

## Ethics Statement

The Human Ethics Review Committee of each institution approved the study protocol.

## Consent

Written informed consent was obtained from all participants. This study was conducted in accordance with the principles of the Declaration of Helsinki.

## Conflicts of Interest

The authors declare no conflicts of interest.

## Supporting information


**Figure S1:** Exercise training. (a) Standing up, (b) knee extension, (c) hip flexion, (d) hip abduction, and (e) heel lift.

**Figure S2:** Hinex Rehadays.

**Table S1:** Changes in body composition and functional capacity during neoadjuvant chemotherapy.

## Data Availability

The authors declare that the data supporting the findings of this study is available in the article and its Supporting Information.
