# Supplementary figures and images for: Exercise and Nutrition Prehabilitation Program During Preoperative Chemotherapy Followed by Esophagectomy in Older Patients With Esophageal Cancer: A Randomized Clinical Trial
Source: Ann Gastroenterol Surg. 2025 Dec 8;10(2):470–82. doi: 10.1002/ags3.70127 (PMC12962008; doi:10.1002/ags3.70127)

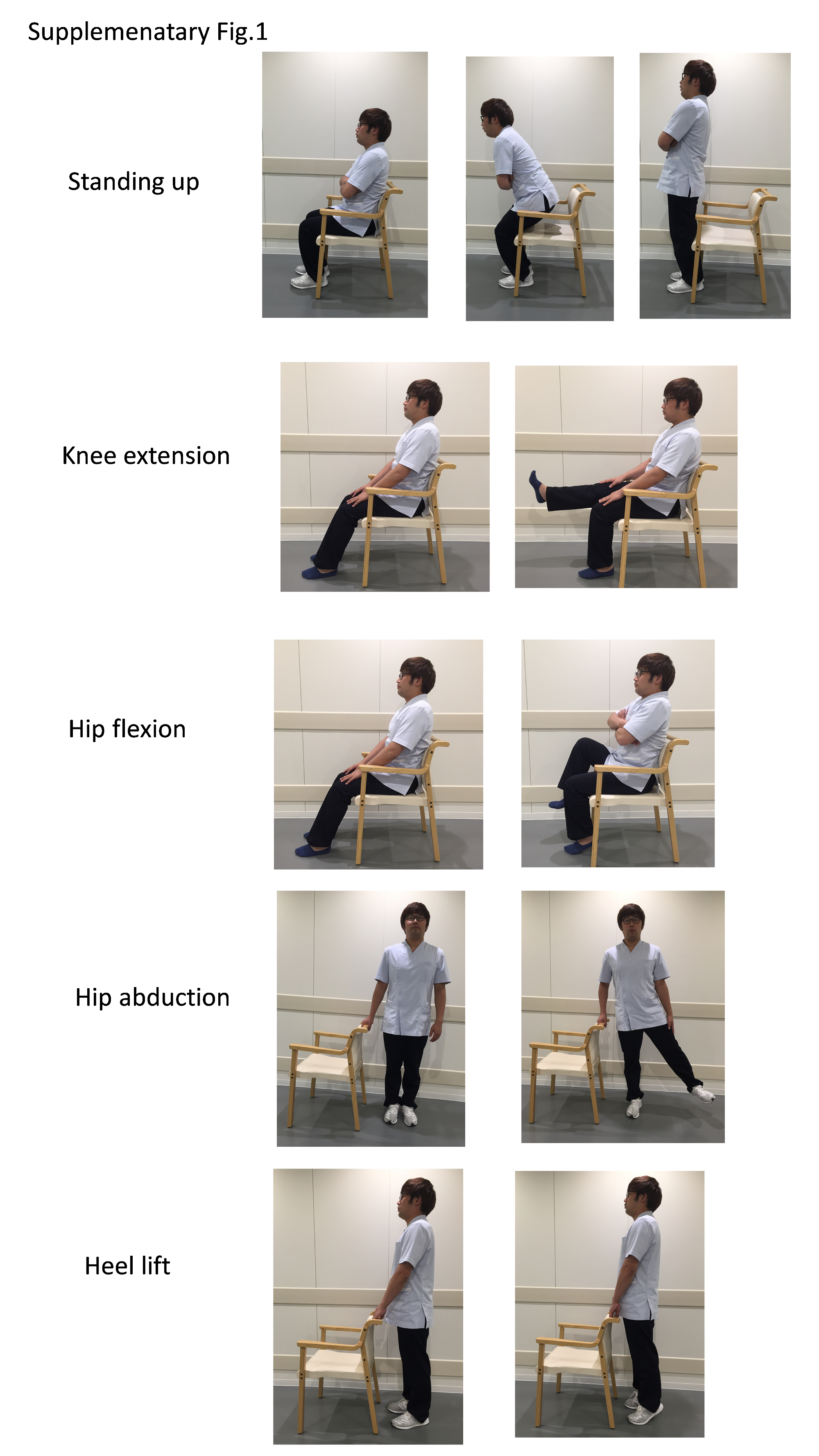

Supplement: Supplementary file 1 — Figure S1: Exercise training. (a) Standing up, (b) knee extension, (c) hip flexion, (d) hip abduction, and (e) heel lift. [file AGS3-10-470-s001.tif]

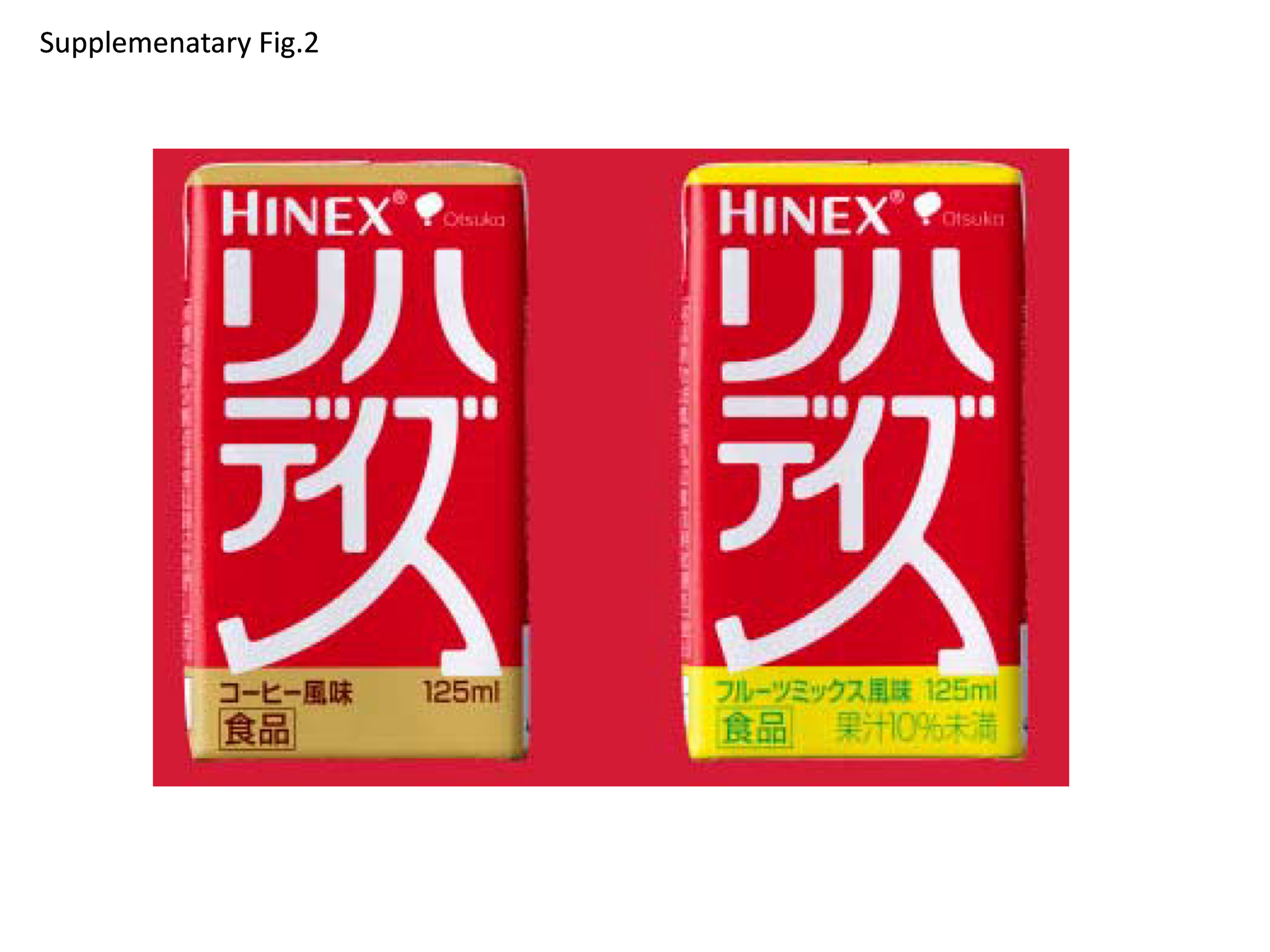

Supplement: Supplementary file 2 — Figure S2: Hinex Rehadays. [file AGS3-10-470-s002.tif]
