# Supplementary material for: Exercise and Nutrition Prehabilitation Program During Preoperative Chemotherapy Followed by Esophagectomy in Older Patients With Esophageal Cancer: A Randomized Clinical Trial
Source: Ann Gastroenterol Surg. 2025 Dec 8;10(2):470–82. doi: 10.1002/ags3.70127 (PMC12962008; doi:10.1002/ags3.70127)
Supplement: Supplementary file 3 — Table S1: Changes in body composition and functional capacity during neoadjuvant chemotherapy. [file AGS3-10-470-s003.docx]

Supplemental Table 1. Changes in body composition and functional capacity during neoadjuvant chemotherapy

|  |  | Group A  (n=31) | Group B  (n=26) | Group C  (n=31) | p (A vs. B)  /p (A vs. C) |
| --- | --- | --- | --- | --- | --- |
| Body weight | Before NAC | 57.8 ±11.2 | 55.0 ±8.7 | 58.2 ±12.1 | 0.149 |
|  | After NAC | 56.6 ±10.6 | 55.1 ±8.8 | 58.8 ±12.1 | 0.014 |
|  | Rate of change | 98.2 ±5.6 | 100.3 ±5.1 | 101.2 ±3.2 |  |
| Skeletal muscle mass | Before NAC | 24.1 ±4.9 | 23.4 ±3.9 | 25.1 ±5.0 | 0.140 |
|  | After NAC | 23.8 ±4.8 | 23.6 ±4.2 | 25.5 ±4.9 | 0.013 |
|  | Rate of change | 98.9 ±5.1 | 100.9 ±4.9 | 101.7 ±3.4 |  |
| Skeletal muscle mass index | Before NAC | 6.8 ±1.1 | 6.7 ±0.8 | 7.1 ±1.2 | 0.096 |
|  | After NAC | 6.8 ±1.1 | 6.9 ±1.0 | 7.4 ±1.2 | 0.044 |
|  | Rate of change | 101.0 ±7.0 | 104.1 ±6.5 | 104.2 ±4.9 |  |
| Handgrip strength | Before NAC | 31.5 ±7.9 | 30.7 ±6.0 | 33.1 ±6.1 | 0.673 |
|  | After NAC | 29.6 ±7.2 | 30.1 ±5.9 | 32.5 ±6.4 | 0.792 |
|  | Rate of change | 97.2 ±12.2 | 98.9 ±12.3 | 98.1 ±8.7 |  |
| Gait speed | Before NAC | 1.36 ±0.25 | 1.37 ±0.19 | 1.42 ±0.21 | 0.760 |
|  | After NAC | 1.35 ±0.27 | 1.35 ±0.19 | 1.55 ±0.22 | 0.031 |
|  | Rate of change | 101.0 ±15.4 | 102.9 ±18.7 | 110.6 ±16.3 |  |
| 6-minute walk distance | Before NAC | 457.3 ±79.3 | 459.0 ±66.1 | 485.2 ±64.5 | 0.768 |
|  | After NAC | 465.8 ±84.7 | 457.7 ±59.0 | 511.3 ±65.5 | 0.342 |
|  | Rate of change | 103.2 ±12.7 | 102.0 ±15.2 | 106.2 ±9.8 |  |
| Albumin | Before NAC | 4.0 ±0.4 | 3.8 ±0.5 | 3.9 ±0.4 | 0.282 |
|  | After NAC | 3.9 ±0.4 | 3.9 ±0.4 | 3.9 ±0.4 | 0.798 |
|  | Δchange | -0.1 ±0.5 | +0.1 ±0.6 | 0.0 ±0.4 |  |
| Lymphocyte | Before NAC | 1401 ±486 | 1443 ±528 | 1350 ±376 | 0.125 |
|  | After NAC | 1587 ±617 | 1448 ±519 | 1523 ±534 | 0.904 |
|  | Δchange | +185 ±406 | +5 ±469 | +173 ±406 |  |
